# Polygenetic-Risk Scores for A Glaucoma Risk Interact with Blood Pressure, Glucose Control, and Carbohydrate Intake

**DOI:** 10.3390/nu12113282

**Published:** 2020-10-26

**Authors:** Donghyun Jee, ShaoKai Huang, Suna Kang, Sunmin Park

**Affiliations:** 1 Division of Vitreous and Retina, Department of Ophthalmology, St. Vincent’s Hospital, College of Medicine, The Catholic University of Korea, Suwon 16247, Korea; doj087@mail.harvard.edu; 2 Food and Nutrition, Obesity/Diabetes Research Center, Hoseo University, Asan 31499, Korea; huangsk0606@gmail.com (S.H.); roypower003@naver.com (S.K.)

**Keywords:** glaucoma, polygenetic-risk scores, gene-gene interaction, carbohydrate intake, gene-nutrient interaction, precision medicine

## Abstract

Glaucoma, a leading cause of blindness, has multifactorial causes, including environmental and genetic factors. We evaluated genetic risk factors of glaucoma with gene-gene interaction and explored modifications of genetic risk with gene-lifestyles interaction in adults >40 years. The present study included 377 subjects with glaucoma and 47,820 subjects without glaucoma in a large-scale hospital-based cohort study from 2004 to 2013. The presence of glaucoma was evaluated by a diagnostic questionnaire evaluated by a doctor. The genome-wide association study was performed to identify genetic variants associated with glaucoma risk. Food intake was assessed using a semiquantitative food frequency questionnaire. We performed generalized multifactor dimensionality reduction analysis to construct polygenetic-risk score (PRS) and explored gene × nutrient interaction. PRS of the best model included LIM-domain binding protein*-2* (*LDB2*) rs3763969, cyclin-dependent kinase inhibitor 2B (*CDKN2B*) rs523096, *ABO* rs2073823, phosphodiesterase-3A (*PDE3A*) rs12314390, and cadherin 13 (*CDH13*) rs12449180. Glaucoma risk in the high-PRS group was 3.02 times that in the low-PRS group after adjusting for confounding variables. For those with low serum glucose levels (<126 mg/dL), but not for those with high serum glucose levels, glaucoma risk in the high-PRS group was 3.16 times that in the low-PRS group. In those with high carbohydrate intakes (≥70%), but not in those with low carbohydrate intakes, glaucoma risk was 3.74 times higher in the high-PRS group than in the low-PRS group. The glaucoma risk was 3.87 times higher in the high-PRS group than in the low-PRS group only in a low balanced diet intake. In conclusion, glaucoma risk increased by three-fold in adults with a high PRS, and it can be reduced by good control of serum glucose concentrations and blood pressure (BP) with a balanced diet intake. These results can be applied to precision nutrition to reduce glaucoma risk.

## 1. Introduction

Glaucoma is the second most common cause of irreversible vision loss worldwide [1]. Glaucoma is the result of optic neuropathies primarily caused by raised ocular pressure, and these neuropathies promote the progressive degeneration of retinal ganglion cells to induce visual loss [1]. Although the major risk factor of glaucoma is increased intraocular pressure, some glaucoma patients have recently been reported to have normal ocular pressure [2]. The risk factors of glaucoma include intraocular pressure, ocular perfusion pressure, ocular blood flow, myopia, central corneal thickness, and optic disc hemorrhages [3]. Increased intraocular pressure is considered to be associated with serum glucose concentrations and blood pressure, but these relations remain controversial. Accumulating evidence has shown that increased intraocular pressure is associated with T-cell-mediated autoimmunity [2]. Low-grade inflammation may initially induce an adaptive reaction of the retina to lead to excessive glial reactions that increase adaptive immune responses. Furthermore, it has been suggested that activated adaptive immunity contributes to progressive neural damage and the development of glaucoma [2].

Elevated intraocular pressure is associated with insulin resistance, which is a common feature of metabolic syndrome [4,5]. However, the components of metabolic syndrome exhibit different associations with intraocular pressure. Of these components, blood pressure is a well-known risk factor of increased intraocular pressure. High fasting glucose levels and obesity are also associated with the increment of intraocular pressure. On the other hand, the association between lipid profiles and intraocular pressure has not been studied. However, in East Asians, few studies have addressed the relationship between glaucoma and metabolic syndrome. Diabetes is an independent risk factor of glaucoma [6,7], which is linked to the neurodegeneration caused by central insulin resistance [8]. Therefore, associations between glaucoma risk and systemic insulin resistance and metabolic syndrome remain unclear due to inconsistencies between reported results.

Genetic and environmental factors influence the incidence of glaucoma. In particular, etiologic studies have demonstrated its incidence is dependent on ethnicity, which implies genetic variations play a critical role in glaucoma. More specifically, primary open-angle glaucoma is associated with inflammation-related genes such as toll-like receptor 4 (*TLR4*) rs4986791 and rs2149356 [9] and interleukin (IL)-10 rs1800871 and rs1800872 [10]. Glaucoma has been reported to be associated with cell-cell adhesion via the activation of transforming growth factor-β signaling [11]. Moreover, since open-angle glaucoma has been linked with insulin resistance, its incidence may have a positive association with insulin/insulin-like growth factor signaling. Some studies have been conducted to determine the effects of lifestyles, including nutrient intake, on glaucoma risk, and vitamin B1 and C and retinol intakes have been reported to be negatively associated with glaucoma risk, whereas magnesium intake is positively associated [12,13]. Interestingly, polyunsaturated fat intake with a higher ratio of *n*-3 and *n*-6 fatty acids has been shown to increase the risk of primary open-angle glaucoma and is suggested to be associated with the production of endogenous prostaglandin F2-α [14]. However, reports on the relation between polyunsaturated fatty acid intake and glaucoma are inconsistent, and smoking cessation, moderate aerobic exercise, a balanced diet, and coffee and tea intake have been shown to protect against the development and progression of primary open-angle glaucoma [15]. These findings demonstrate that environmental factors can reduce the risk of glaucoma. Furthermore, it is evident that environmental factors interact with genetic factors and that these gene-environmental interactions have potential utility in precision medicine.

Here, we aimed to explore the polygenetic variants of glaucoma risk related to inflammation and insulin resistance concerning gene-gene interactions and polygenetic variant and lifestyle interactions in the middle-aged and elderly individuals.

## 2. Materials and Methods

### 2.1. Participants Recruitment

During 2004–2013, a total of 20,274 men and 38,371 women (total 58,645) aged >40 years voluntarily participated in hospital-based city cohort studies (the Korean Genome and Epidemiology Study (KoGES)) organized by the Korean Center for Disease and Control. All procedures of the KoGES were performed according to the Declaration of Helsinki, and they were approved by the Institutional Review Board of the Korean National Institute of Health for the KoGES (KBP-2015-055) and Hoseo University (1041231-150811-HR-034-01). All subjects that participated in KoGES provided written informed consent.

### 2.2. Criteria of Glaucoma

Participants were asked whether they had received a diagnosis of glaucoma from a physician, and those that answered affirmatively were considered to have primary glaucoma. A total of 10,448 participants did not answer the question about the glaucoma diagnosis, and they were eliminated in the analysis.

### 2.3. Anthropometric and Biochemical Measurements

Information on age, education, income, smoking history and alcohol consumption, and physical activity was collected during a health interview. Education level was divided into three groups: less than high school, high school, and college or more. Household income (USD/month) was categorized as very low (<$1000), low ($1000–$2000), intermediate ($2000–$4000), and high (>$4000) [16]. Smoking status was divided into three categories: current smoker, past smoker, and never-smoker [16]. According to average daily alcohol consumption, the participants were categorized into nondrinker, light drinker (0–1 g), moderate drinker (1–20 g), and heavy drinker (>20 g) (Table 1) [16]. Dairy product consumptions (milk, yogurt, and cheese) were also obtained.

Body weight, height, and waist circumference were measured using a standardized procedure [17]. Body mass index (BMI) was calculated by dividing weight in kilograms by the height (in meters) squared. Blood was collected after an overnight fast, and plasma and serum samples were used for biochemical measurements [17]. Fasting serum glucose and blood hemoglobin A1c (HbA1c; glycated hemoglobin) concentrations were determined using a Hitachi 7600 Automatic Analyzer (Hitachi, Tokyo, Japan). Blood pressure was measured on the right arms at the heart level in a sitting position.

### 2.4. Assessment of Food and Nutrient Intakes Using a Semiquantitative Food Frequency Questionnaire (SQFFQ)

Dietary intakes were estimated using an SQFFQ developed and validated for the KoGES [18]. This questionnaire requested information about the consumption of specified food items. The participants completed the SQFFQ. Based on the consumption of 103 food items, daily nutrient intakes were calculated using data from a food intake nutrient database maintained by the Korean Nutrition Society and the Computer-Aided Nutritional Analysis Program (CAN Pro) 3.0 (Seoul, Korea) [18].

### 2.5. Genotyping and Quality Control

Genotype data were provided by the Center for Genome Science, Korea National Institute of Health. Genomic DNA was extracted from whole blood, and genotypes were determined using the Affymetrix Korean Chip (Affymetrix, Santa Clara, CA, USA) made available for scientific studies. This chip has been previously used to study Korean genetic variants and included disease-related single-nucleotide polymorphism (SNP) [19]. Genotyping accuracy of the SNP results was examined by Bayesian robust linear modeling using the Mahalanobis distance (BRLMM) genotyping algorithm [20]. The genotype results met genotyping accuracy of ≥98%, a missing genotype call rate of <4%, heterozygosity of <30%, and show no gender bias. Genetic variants used for further analysis met the Hardy-Weinberg equilibrium (HWE) (*p* > 0.05).

### 2.6. Identification of the Best Model for Gene-Gene Interactions by Generalized Multifactor Dimensionality Reduction (GMDR) Method among the Genetic Variants Selected by Logistic Regression

**Figure 1:**
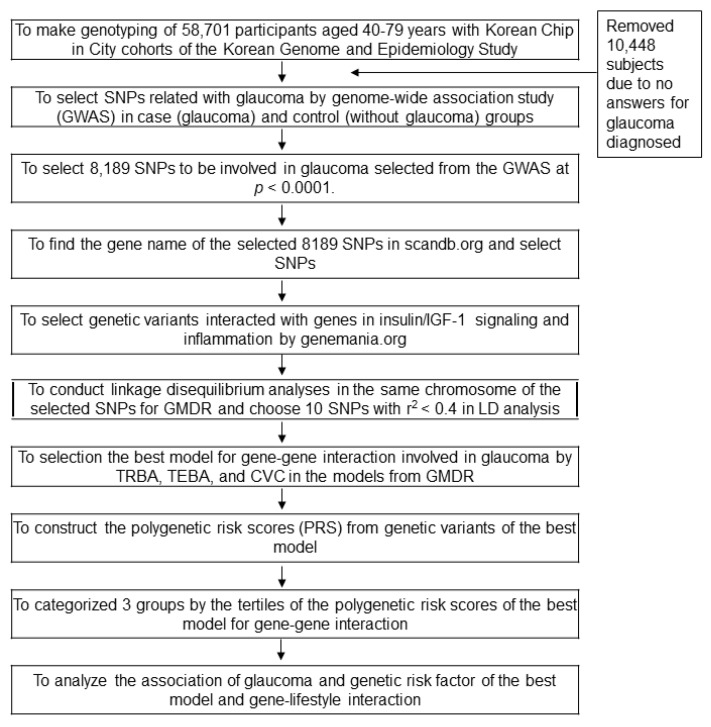
The flow chart to make polygenetic-risk scores to influence glaucoma risk. GMDR, generalized multifactor dimensionality reduction; TRBA, trained balanced accuracy; TEBA, testing balanced accuracy; CVC, cross-validation consistency.

A flow chart of the procedure used to calculate polygenetic-risk scores (PRSs) of glaucoma risk is presented in Figure 1.

Participants were categorized as having glaucoma (*n* = 377) or not (*n* = 47,820); 10,448 participants did not answer the question, and they were excluded. Logistic regression was performed to identify genetic variants associated with glaucoma risk (*p* < 0.0001). Corresponding genes were identified using scandb.org. Genes that interacted with insulin/insulin like-growth factor-1 (IGF-1) signaling related to insulin resistance and inflammation that influence glaucoma risk were selected using genemania.org. The 43 selected SNPs were subjected to generalized multifactor dimensionality reduction (GMDR) analysis to explore gene-gene interactions associated with glaucoma risk. Linkage disequilibrium (LD) analyses were performed on selected genetic variants in the same chromosome using Haploview 4.2 in PLINK (Boston, MA, USA). SNPs showing high LDs (*r*^2^ ≥ 0.4) were excluded as they provided similar information on glaucoma risk. The best model for predicting gene-gene interactions that influence glaucoma risk was selected by trained balanced accuracy (TRBA), testing balanced accuracy (TEBA), and cross-validation consistency (CVC) using GMDR. The final 10 potential genetic variants in the same chromosome included in the best model did not have a strong correlation in LD (*r*^2^ < 0.4). PRSs for the best model were calculated by summing the number of risk alleles for each selected SNP in the best gene-gene interaction model. PRSs were divided into three categories by tertile; a high PRS indicated the individual concerned had a high number of risk alleles.

### 2.7. Statistical Analyses

Statistical analysis was performed using PLINK v. 1.9. (http://zzz.bwh.harvard.edu/plink/; Boston, MA, USA) and SAS (v. 9.3.; SAS Institute, Cary, NC, USA). The best gene-gene interaction model was selected using a GMDR program and the signed-rank test using a *p*-value of <0.05 with TRBA and TEBA with or without adjusting for covariates of age, gender, education, income level, and body mass index [21]. Ten-fold cross-validation was used to check CVC since the sample size was greater than 1000 [21]. From the best model determined by GMDR analysis, the number of the risk allele in each SNP was counted in the selected best model [22]. For example, when the G allele had a positive association with an increased risk of glaucoma, TT, GT, and GG were assigned scores of 0, 1, and 2, respectively. PRSs were calculated by summing risk allele scores for each SNP included in the PRS for the best model.

The descriptive statistics of categorical variables (e.g., gender and lifestyle) were calculated by PRS tertile and designated as low, middle, or high. Frequency distributions of classification variables were analyzed using the chi-squared test. Means and standard deviations were calculated for continuous variables according to the PRS categories, and significant differences between groups were determined by one-way analysis of variance (ANOVA) with or without adjustment for covariates. Tukey’s test was used to performed multiple group comparisons.

The association between PRSs and glaucoma risk was investigated using logistic regression analysis after adjusting for covariates. Odds ratios (ORs) and 95% confidence intervals (CI) were analyzed using logistic regression analysis with low-PRS as a reference, after either adjusting for age, gender, residence area, survey year, BMI, education, job, and income, or by adjusting age, gender, residence area, survey year, smoking status, alcohol intake, education, job, income, energy intake, physical activity, hypertension, milk intake, percent fat intake, percent carbohydrate intake, and arthritis and dermatitis medicine intake. To determine the interaction between PRSs and lifestyles and dietary intakes, participants were categorized into higher or lower intake groups using the criteria defined by 50th percentiles of each variable. A multivariate interaction model was used to evaluate interactions between PRSs and lifestyles and dietary intake after adjustment for covariates. *p*-values of ≤0.05 were considered statistically significant.

## 3. Results

### 3.1. General Characteristics of Participants with Glaucoma

Participants with glaucoma (*n* = 377) were older than those without glaucoma (*n* = 47,820). Participants aged ≥55 years old had a 3.3-fold greater risk of glaucoma than those aged <55 years old (Table 1), and men had a 1.8-fold higher risk than women (Table 1). Participants with a college degree and a family income of >$2000/month had a lower prevalence of glaucoma than those with less education and a lower income. However, education and income status were not significantly associated with glaucoma risk after adjusting for covariates (gender, age, residence area, surveyed year, BMI, smoking, alcohol, education, job, income, energy intake, and arthritis and dermatitis medicine intake) (Table 1). Furthermore, daily regular exercise and alcohol intake had no significant association with glaucoma risk after adjusting covariates, and coffee, energy, and nutrient intakes were similar in those with or without glaucoma (Table 1).

### 3.2. Association between Glaucoma Risk and Metabolic Syndrome

Interestingly, participants with glaucoma had a 1.4-fold higher risk of metabolic syndrome than those without glaucoma (Table 2). Regarding components of metabolic syndrome, fasting serum glucose concentrations were significantly associated with glaucoma risk, but blood pressure, serum lipid, and waist circumference were not (Table 2). Fasting serum glucose concentration and glycated hemoglobin (HbA1C) were 1.5- and 1.7-fold higher, respectively, in those with glaucoma. Moreover, serum C-reactive protein-1 concentrations (>0.8 mg/dL) increased glaucoma risk by 2.1-fold in those with glaucoma (Table 2).

### 3.3. Selection of Genetic Variants Associated with Glaucoma Risk Using GMDR

Using the genetic variants selected for glaucoma risk by logistic regression, we used GMDR to investigate gene-gene interactions for genetic variants. Ten genetic variants were included in the GMDR analysis. The genetic characteristics of these 10 SNPs are presented in Table 3. Adjusted ORs of seven SNPs were significantly greater than 1 and those of three SNPs were between 0 and 1 (Table 3). All selected SNPs satisfied HWE criteria (*p* > 0.05).

Of the 10 SNPs, the best model for gene-gene interactions included five genetic variants, including LIM-domain binding protein 2 (*LDB2*) rs3763969, cyclin-dependent kinase inhibitor *2B* (*CDKN2B*) rs523096, *ABO* rs2073823, phosphodiesterase 3A (*PDE3A*) rs12314390, and cadherin 13 (CDH13) rs12449180 after adjusting for age and gender (adjustment 1) and age, gender, survey year, residence area, BMI (adjustment 2; Table 4). The model including these five SNPs had a TRBA of 0.6274 and a TEBA of 0.5927 after adjusting for age, gender, survey year, residence area, and BMI (adjustment 2; *p* < 0.001), and CVC was 10/10 (Table S1). The TRBA, TEBA, and CVC in model 1 with the adjusting for age and gender were similar to those in model 2 with adjustment of more covariates. In addition, the model including the 10 SNPs had 0.7603 and 0.5283 of TRBA and TEBA, respectively, and CVC was 10/10 (*p* < 0.001) after adjusting for covariates. The results indicated these 5 and 10 genetic variants exhibited gene-gene interactions that increased glaucoma risk. Since the model including five SNPs met the significant gene-gene interaction with fewer SNPs, the model with five SNPs was better for showing genetic impact for glaucoma risk.

### 3.4. A Positive Association between PRSs and Glaucoma Risk

PRS for the best model was generated and used to quantify the genetic risk of glaucoma. A high-PRS increased glaucoma risk by 2.9- and 3.0-fold in models 1 and 2, respectively (Table 4). Adjustment 1 and 2 in the best model included different covariates: adjustment 1 included age, gender, residence area, survey year, BMI, education, job, and income as covariates, and adjustment 2 included age, gender, residence area, survey year, smoking, alcohol, education, job, income, energy, activity, hypertension, milk, fat percent intake, carbohydrate percent intake, and arthritis and atopic dermatitis medicine intake. However, a high-PRS had no association with cataract risk (Table 4). PRSs were not associated with the risk of metabolic syndrome or blood pressure in adjustment 1 or 2. Interestingly, a medium-PRS, but not a high-PRS, was negatively associated with type 2 diabetes since SNPs for the best model were selected from the insulin/IGF-signaling pathway. These results showed that a high-PRS had a positive association with glaucoma risk.

### 3.5. Interaction between PRSs and Lifestyle in Glaucoma Risk 

**Figure 2:**
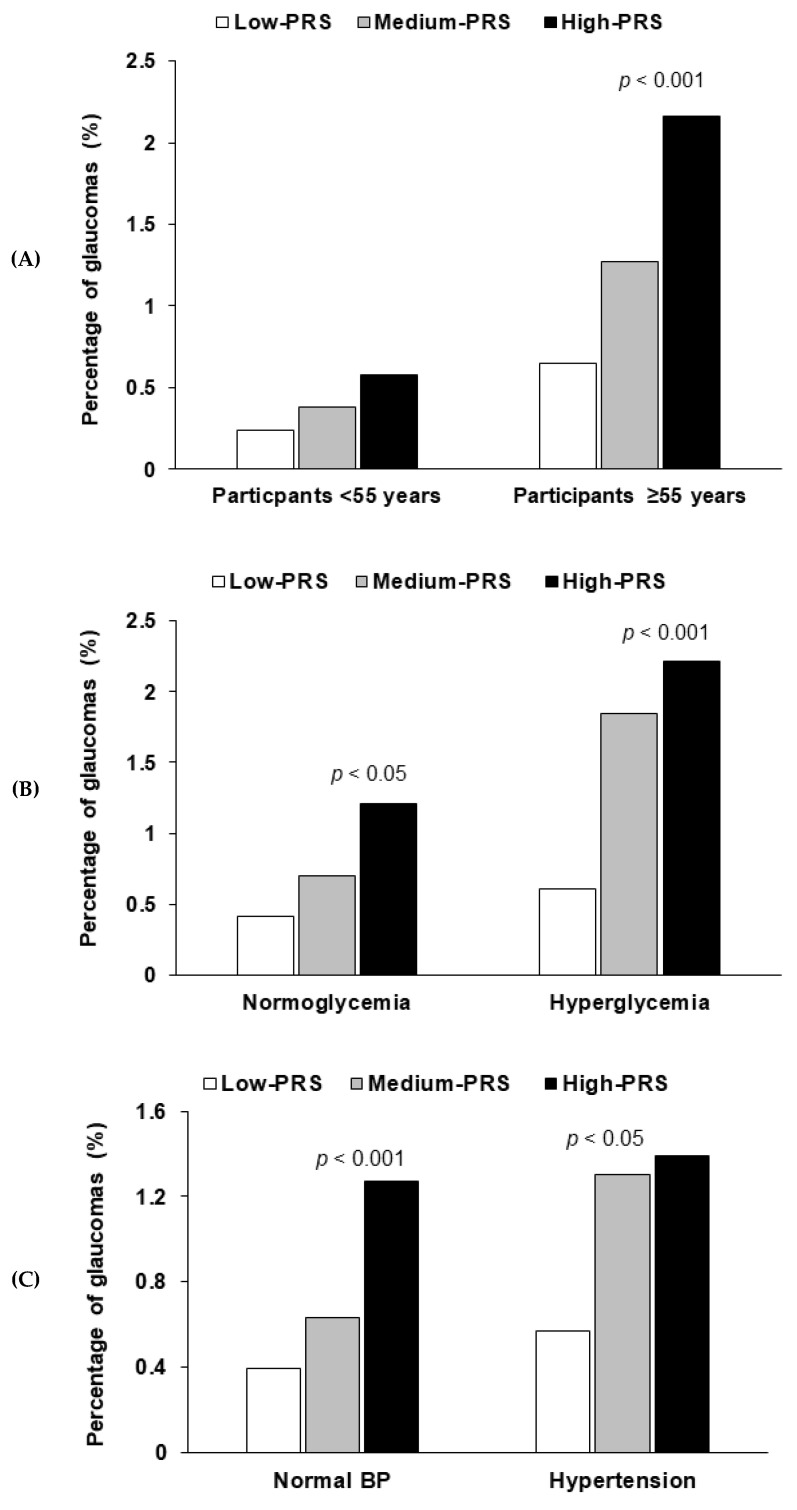
The frequency distribution of glaucoma in the three groups of polygenetic-risk scores (PRS) of the best model including *LDB2* rs3763969, *CDKN2B* rs523096, *ABO* rs2073823, *PDE3A* rs12314390, and *CDH13* rs12449180 according to the metabolic status. (**A**) According to age (cutoff point: 55 years old). (**B**) According to serum glucose concentrations (cutoff point: 126 mg/dL serum glucose concentrations). (**C**) According to the blood pressure (cutoff point: 130 mmHg for systolic blood pressure (SBP) and 90 mmHg for diastolic blood pressure (DBP)). PRS was calculated by the summation of each genetic-risk score of the best model, and PRS was categorized into three groups by the tertiles (Low-PRS, Medium-PRS, and High-PRS). BP, blood pressure.

Age showed an interaction with PRSs to glaucoma risk. In participants aged ≥55 years, a high-PRS had a much higher glaucoma risk than a low-PRS, but this was not observed in participants aged <55 years (Table 5). The prevalence of glaucoma was much greater for those with a high-PRS than for those with a low-PRS only in participants ≥55 years old (Figure 2A). Blood pressure (*p* = 0.011) and hyperglycemia (*p* = 0.46) interacted with PRS (Table 5). A high-PRS was associated with greater glaucoma risk than a low-PRS, particularly in participants with low blood pressure and normoglycemia (Table 5). Glaucoma incidence was greater in a high-PRS than in a low-PRS in all participants, but the incidence was much greater in the participants with hyperglycemia and hypertension (Figure 2B,C).

**Figure 3:**
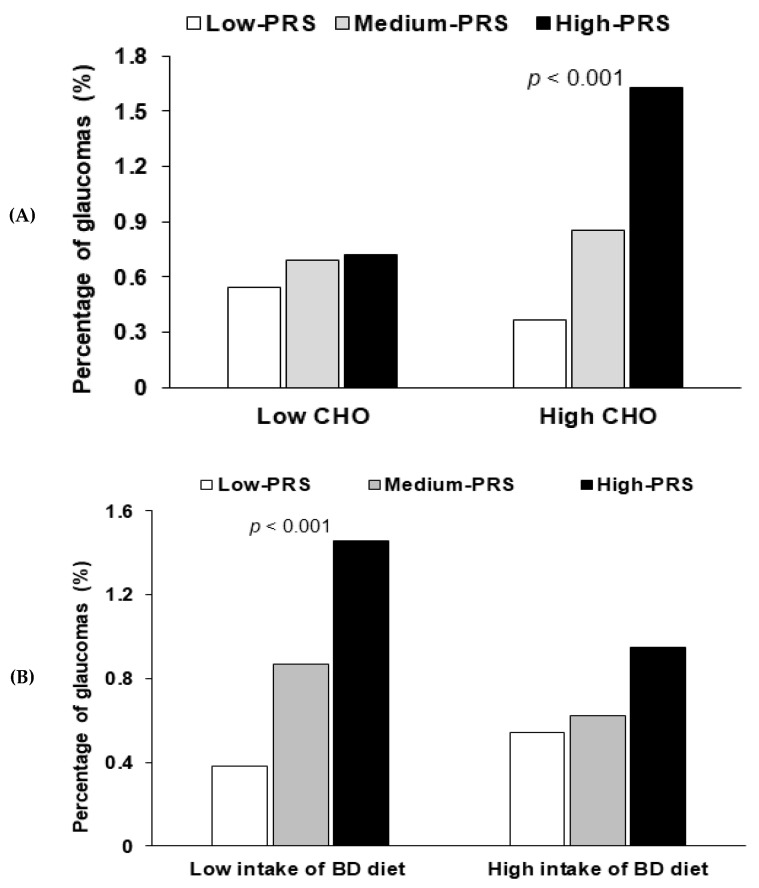
The frequency distribution of glaucoma in the three groups of polygenetic-risk scores (PRS) of the best model including *LDB2* rs3763969, *CDKN2B* rs523096, *ABO* rs2073823, *PDE3A* rs12314390, and *CDH13* rs12449180 according to the nutrient and food intake. (**A**) According to the carbohydrate intake (cutoff point: 70 energy %). CHO, carbohydrate. (**B**) According to the intake of a balanced diet pattern (cutoff point: 70th percentile). PRS was calculated by the summation of polygenetic-risk scores of the best model, and PRS was categorized into three groups by the tertiles (Low-PRS, Medium-PRS, and High-PRS). BD, balanced diet.

For energy and nutrient intakes, only carbohydrate intake showed an interaction with PRS in terms of glaucoma risk (*p* = 0.008; Table 5). No interact was found for energy intake (*p* = 0.155), protein (*p* = 0.205), fat (*p* = 0.185), or Na intake (*p* = 0.792). For participants with high carbohydrate intake, glaucoma risk was 3.7-fold higher for those with a high-PRS than for those with a low-PRS (Table 5), and in those with high carbohydrate intakes, the prevalence of glaucoma was much higher among those with a high-PRS (Figure 3A). There was no interaction of PRS with coffee (*p* = 0.687) and alcohol intake (*p* = 0.457). No interaction was evident between daily regular exercise and PRSs for glaucoma risk (*p* = 0.095; Table 5).

A balanced dietary (BD) pattern explained 40.1% of Korean diet patterns in three different dietary patterns. A BD pattern includes the consumption of beans, potatoes, kimchi, green and white vegetables, mushrooms, fatty and white fish, seaweeds, fruits, and pickles (loading ≥0.4). A BD pattern had an interaction with glaucoma risk (*p* = 0.046). A low BD pattern intake had a positive association by 3.87-fold with PRS (*p* < 0.0001), but a high BD pattern intake did not have a significant association with PRS (Table 5). Glaucoma prevalence was much higher in participants with a low BD intake and a high-PRS than in those with a low-PRS (Figure 3B). It suggested that adults with high-PRS needed to consume a high BD pattern to reduce the glaucoma risk.

## 4. Discussion

In the present study, we constructed a glaucoma PRS model by generalized multifactor dimensionality reduction and evaluated whether genetic impact represented by PRS was associated with the presence of glaucoma. Moreover, we assessed gene-nutrient interactions by evaluating the effect of nutrition on the risk of glaucoma using PRSs. The risk of glaucoma was found to be 3.01 times higher in participants with a high-PRS than in those with a low-PRS after adjusting for covariates. The genetic risk of glaucoma was modified by serum glucose levels, carbohydrate intakes, and consumption of a balanced diet. More specifically, the genetic risk of glaucoma was significantly higher in participants with low blood pressure, low serum glucose, high carbohydrate intake, and low balanced diet intake.

High levels of fasting serum glucose and HbA1c had a higher association with the risk of glaucoma by 1.53 and 1.66 times, respectively. High serum glucose and HbA1c are well-known risk factors of glaucoma. A meta-analysis involving 47 studies and 2,981,342 individuals from 16 countries demonstrated that the pooled relative risk of glaucoma was 1.48 in patients with diabetes as compared with normal controls [7]. Another cross-sectional study that included 3229 individuals involved in a National Health and Nutritional Examination Survey reported that diabetes was strongly associated with the prevalence of glaucoma (OR = 2.12) [23]. However, in the present study, low serum glucose levels were found to increase the genetic risk of glaucoma, and in participants with a low serum glucose level, those with a high-PRS had a 3.165 times higher risk of glaucoma than those with a low-PRS, whereas no such difference was observed for participants with a high serum glucose level. This finding suggests that the genetic risk of glaucoma is elevated in those with a low serum glucose level.

The genetic risk of glaucoma was significant only in participants with high carbohydrate intake, indicating that there was no significant genetic risk for glaucoma in subjects with low carbohydrate intake. A recent case-control study involving 37 glaucoma patients and 36 controls showed that carbohydrate ingestion induced autonomic dysregulation, which may lead to the development of glaucoma [24]. One possible explanation for this finding is that high carbohydrate intake might enhance the genetic risk of glaucoma development through autonomic dysregulation. A further longitudinal epidemiologic study is warranted to confirm that individuals with a high carbohydrate intake are more susceptible to glaucoma development.

We found that the genetic risk of glaucoma was significant only in subjects with a low balanced diet intake. The risk of glaucoma for those with a low balanced diet intake was 3.87 times higher in the high-PRS group than in the low-PRS group, whereas the risk of glaucoma for those with a high intake of a balanced diet was not significantly different in these two groups. Several studies have demonstrated that a balanced diet, including green leafy vegetables, omega fatty acids, and moderate intake of hot tea and coffee, possibly protects against the development or progression of glaucoma [15]. We suggest this finding is due to the protective effects of a high balanced intake attenuating the effect of genetic risk on glaucoma development.

We compared genetic impacts for glaucoma and cataract using PRSs. The risk of glaucoma in participants with a high-PRS was 3.02 times higher than in those with a low-PRS. However, the risk of cataract was not significantly different in these groups. One possible explanation for this finding is that glaucoma development is more dependent on genetic risk than cataract development. Age-related cataract development is mainly caused by environmental factors such as sunlight exposure, lens aging, inflammation, and oxidation. These factors are all associated with the free-radical generation, which leads to the aggregation of lens proteins and subsequent cataract formation [15,25,26].

The present study has several strengths and limitations. The strength of this study is that it involved a large number of participants, that is, 377 patients with glaucoma and 47,820 subjects without, which undoubtedly increased study reliability. This study could demonstrate a new scenario of therapeutic approaches to counteract the potential genetic risk of glaucoma development by the control of lifestyle. However, it also has several limitations. First, the presence of glaucoma was diagnosed in an individual by a doctor, and a questionnaire was used to answer if glaucoma was present. However, it was not reevaluated for the type of glaucoma by an ophthalmologist for the present study. Second, the study is inherently limited by its cross-sectional design, which prevented our inferring causality. However, it is unlikely that the presence of glaucoma changed eating patterns. Finally, the SQFFQ method used to evaluate usual food intake also has limitations, and at the individual level, nutrition intakes might be overestimated, because data was dependent on participant recall. Nevertheless, the SQFFQ was developed and validated for KoGES and has been widely utilized [18,27,28].

In conclusion, we constructed PRS for glaucoma by generalized multifactor dimensionality reduction and found that the risk of glaucoma was 3.01 times higher for the participants with a high-PRS. The present study demonstrates the existence of a nutrition-gene interaction in the risk of glaucoma development. Such genetic risk with high-PRS can be reduced by good control of serum glucose concentrations and blood pressure with a balanced diet intake. The results can be applied for precision medicine to protect against glaucoma in the person with genetic risk. Given that glaucoma is the leading cause of blindness worldwide, further study is required to evaluate nutrition-gene interactions to reduce the risk of glaucoma development.

## Figures and Tables

**Table 1:** Socioeconomic and lifestyle characteristics of the participants according to glaucoma presence.

Parameters Related to Glaucoma	Non-Glaucoma (*n* = 47,820)	Glaucoma (*n* = 377)	Adjusted OR for Glaucoma Risk (OR, 95% CI)
Age (years) ^1^	53.7 ± 5.5	58.2 ± 5.4 ^***^	3.325 (2.623–4.213) ^***^
Gender (number, male %)	16,193 (33.9)	171 (45.4) ^***^	1.797 (1.430–2.257) ^***^
Education (number, %)			
<High school	6417 (18.5)	66 (25.7) ^**^	1
High school	7689 (22.1)	60 (23.4)	1.060 (0.724–1.552)
College more	20,619 (59.4)	131 (51.0)	1.169 (0.821–1.665)
Income (number, %)			
<$1000/m	4508 (9.94)	66 (18.5) ^***^	1
$1000–$2000	9722 (21.4)	78 (21.9)	0.699 (0.496–0.986)
$2000–$4000	20,047 (44.2)	133 (37.4)	0.756 (0.542–1.053)
>$4000	11,084 (24.4)	79 (22.2)	0.895 (0.607–1.320)
Exercise (number, %)			
No	21,531 (45.2)	145 (38.7) ^*^	1
Yes	26,144 (54.8)	230 (61.3)	1.216 (0.971–1.523)
Alcohol intake (number, %)			
No	27,131 (56.7)	238 (63.1) ^*^	1
Mild drink (0–20 g)	1048 (2.19)	5 (1.33)	0.656 (0.269–1.600)
Moderate drink (≥20 g)	19,641 (41.1)	134 (35.5)	0.789 (0.612–1.016)
Coffee intake (number, %)			
Low (<3 cups/week)	18,037 (37.7)	156 (41.4)	1
Medium (3–16 cups/week)	29,329 (61.3)	218 (57.8)	1.030 (0.767–1.383)
High (≥16 cups/week)	454 (0.95)	3 (0.80)	0.903 (0.704–1.157)
Energy intake ^2^ (kcal)	1743 ± 531	1719 ± 516	0.848 (0.674–1.067)
CHO percent intake ^3^	71.7 ± 20.8	71.6 ± 20.0	1.037 (0.816–1.317)
Fat percent intake ^4^	13.9 ± 8.7	14.1 ± 8.0	1.137 (0.901–1.435)
Protein percent intake ^5^	13.4 ± 5.8	13.3 ± 5.6	0.919 (0.699–1.209)

The values represent means ± standard deviations or number of the subjects (percentage of each group). The cutoff points of the parameters were as follows: ^1^ <55 years old, ^2^ <estimated energy intake, ^3^ <70 energy % of carbohydrate (CHO), ^4^ <15 energy % fat, and ^5^ <15 energy % protein. Adjusted odds ratio (ORs) after adjusting for covariates including gender, age, residence area, surveyed year, body mass index (BMI), smoking, alcohol, education, job, income, energy intake, and arthritis and dermatitis medicine intake in logistic regression models. * Significant differences by cataract at *p* < 0.05, ^**^ at *p* < 0.01, ^***^ *p* < 0.001.

**Table 2:** The association of glaucoma risk and metabolic syndrome.

Components for Metabolic Syndrome	Non-Glaucoma (*n* = 47,820)	Glaucoma (*n* = 377)	Adjusted OR for Glaucoma Risk (OR, 95% CI)
Metabolic syndrome ^1^ (number, %)	6673 (14.0)	81 (21.5) ^***^	1.361 (1.032–1.793) ^#^
BMI ^2^ (kg/m^2^)	23.9 ± 2.8	23.8 ± 2.9	0.927 (0.736–1.167)
Waist circumferences ^3^ (cm)	80.6 ± 8.7	80.8 ± 8.4	0.971 (0.751–1.255)
Serum glucose ^4^ (mg/dL)	95.0 ± 20.2	100.1 ± 26.5 ^***^	1.539 (1.182–2.003) ^##^
Blood HbA1c ^5^ (%)	5.7 ± 0.7	5.9 ± 0.9 ^***^	1.663 (1.170–2.364) ^##^
Serum total cholesterol ^6^ (mg/dL)	197 ± 36	195 ± 38	1.108 (0.867–1.417)
Serum HDL ^7^ (mg/dL)	54.4 ± 13.3	53.7 ± 12.8	1.181 (0.929–1.501)
Serum TG ^8^ (mg/dL)	125 ± 86	119 ± 73	1.134 (0.901–1.428)
Serum BP ^9^ (number, %)	11,627 (24.3)	138 (36.6) ^***^	1.225 (0.968–1.551)
Serum CRP-1 ^10^ (mg/dL)	0.14 ± 0.38	0.18 ± 0.45	2.066 (1.221–3.496) ^##^

The values represent adjusted means ± standard deviations. Adjusted and means odds ratio (ORs) after adjusting for covariates including gender, age, residence area, surveyed year, body mass index (BMI), smoking, alcohol, education, job, income, energy intake, and arthritis and dermatitis medicine intake in logistic regression models. The reference of cutoff points in the parameters were as follows: ^1^ no metabolic syndrome, ^2^ <25 kg/m^2^ body mass index (BMI); ^3^ <90 and 85 cm waist circumferences for men and women, respectively; ^4^ <126 ml/dL fasting serum glucose plus diabetic drug intake; ^5^ <6.5% glycated hemoglobin (HbA1c) plus diabetic drug intake; ^6^ <230 mg/dL serum total cholesterol concentrations; ^7^ ≥40 and ≥50 mg/dL serum high density lipoprotein (HDL) cholesterol concentrations for men and women, respectively, and lipid-lowering drug; ^8^ <150 mg/dL serum triglyceride (TG) concentrations; ^9^ <130 mmHG systolic blood pressure (BP) and <90 mmHg diastolic BP and taking BP-lowering drug; ^10^ <0.8 mg/dL high-sensitive C-reactive protein (CRP-1). * Significantly different between the Non-glaucoma and Glucoma groups at *** *p* < 0.001. ^#^ Significantly different from the Non-glaucoma in multivariate logistic regression at *p* < 0.05, ^##^ at *p* < 0.01.

**Table 3:** The characteristics of the ten genetic variants of genes related to glaucoma risk used for the generalized multifactor dimensionality reduction analysis.

CHR ^1^	SNP ^2^	Location	Mi ^3^	OR ^4^	*p*-Value for OR ^5^	Genes	Feature	MAF ^6^	HWE ^7^
4	rs3763969	16648246	T	0.63 (0.48–0.83)	9.7.E–04	*LDB2*	intron	0.120	0.082
7	rs1852542	42096521	T	1.67 (1.24–2.25)	6.6.E–04	*GLI3*	intron	0.041	0.559
8	rs1020236	135543194	C	1.48 (1.19–1.83)	4.4.E–04	*ZFAT*	intron	0.093	0.732
9	rs523096	22019129	G	0.73 (0.57–0.93)	1.0.E–02	*CDKN2B*	intron	0.134	0.972
9	rs2073823	136132516	A	1.33 (1.13–1.56)	7.6.E–04	*ABO*	intron	0.215	0.941
12	rs12314390	20597977	T	1.70 (1.29–2.25)	1.8.E–04	*PDE3A*	intron	0.048	0.558
13	rs7335337	38221067	G	1.78 (1.34–2.37)	7.8.E–05	*TRPC4*	intron	0.041	0.230
15	rs1319859	99230263	G	1.32 (1.13–1.53)	3.5.E–04	*IGF1R*	intron	0.300	0.226
16	rs12449180	83547527	G	1.42 (1.18–1.69)	1.3.E–04	*CDH13*	intron	0.162	0.162
18	rs3902981	12658191	G	0.73 (0.62–0.87)	3.6.E–04	*SPIRE1*	near-gene-5	0.300	0.492

^1^ Chromosome; ^2^ Single-nucleotide polymorphism; ^3^ Minor allele; ^4^ Odds ratio and lower and upper ends of 95% confidence interval; ^5^ *p*-value for OR after adjusting for age, gender, residence area, survey year, body mass index, daily energy intake, education, and income; ^6^ Minor allele frequency; ^7^ Hardy-Weinberg equilibrium.

**Table 4:** Adjusted odds ratios for glaucoma, age-related cataract, and metabolic syndrome according to the polygenetic risk scores (PRS) of the best model for gene-gene interaction after covariate adjustments.

Glaucoma-Related Diseases	Adjustment 1			Adjustment 2	
	Low-PRS (*n* = 9245)	Medium-PRS (*n* = 31,227)	High-PRS (*n* = 6015)	Medium-PRS (*n* = 31,227)	High-PRS (*n* = 6015)
Glaucoma	1	1.814 (1.280–2.573)	2.937 (1.965–4.389) ***	1.815 (1.213–2.715)	3.021 (1.898–4.809) ***
Cataract	1	0.871 (0.767–0.988)	0.935 (0.782–1.117)	0.898 (0.771–1.045)	0.983 (0.793–1.218)
Metabolic syndrome	1	0.992 (0.924–1.064)	1.004 (0.910–1.108)	0.984 (0.891–1.086)	1.037 (0.903–1.192)
Type 2 diabetes	1	0.879 (0.805–0.959) *	0.951 (0.841–1.075)	0.873 (0.799–0.954) *	0.930 (0.821–1.053)
Blood pressure	1	1.006 (0.949–1.067)	0.997 (0.919–1.082)	0.979 (0.908–1.054)	0.993 (0.894–1.103)

Values represent odd ratios and 95% confidence intervals. PRS was divided into three categories (0–3, 4–5, and >6) by tertiles as the low, medium, and high groups, respectively. Low-PRS was the reference for both model 1 and model 2. * Significantly different from low-PRS in logistic regression analysis at * *p* < 0.05, ** *p* < 0.01, *** *p* < 0.001. Adjustment 1: adjusted for age, gender, residence area, survey year, body mass index (BMI), education, job, and income. Adjustment 2: adjusted for age, gender, residence area, survey year, smoking, alcohol, education, job, income, energy, activity, hypertension, milk, fat percent intake, carbohydrate percent intake, and arthritis and atopic dermatitis medicine intake. * Significantly different from the Low-PRS in multivariate logistic regression at *p* < 0.05, *** at *p* < 0.001.

**Table 5:** Adjusted odds ratios for the glaucoma risk by polygenetic risk scores (PRS) of the best model and gene-environmental interactions after covariate adjustments.

Parameters for Glaucoma Risk	Low-PRS (*n* = 14,420)	Medium-PRS (*n* = 21,641)	High-PRS (*n* = 4201)	Gene-Nutrient Interaction *p*-Value
Less aged people More aged people ^1^	1	1.575 (0.737–3.364) 1.907 (1.185–3.068)	2.577 (1.063–6.246) ^*^ 3.187 (1.844–5.506) ^***^	0.0092
Low BP High BP ^2^	1	1.695 (1.024–2.805) 1.999 (1.021–3.915)	3.659 (2.092–6.399) ^***^ 1.751 (0.723–4.242)	0.0106
Low serum glucose High serum glucose ^3^	1	1.791 (1.149–2.719) 2.020 (0.770–5.297)	3.165 (1.907–5.251) ^***^ 2.195 (0.656–7.337)	0.0460
Low energy intake High energy intake ^4^	1	2.456(1.404–4.293) 1.210(0.669–2.191)	3.959 (2.113–7.417) ^***^ 1.432 (0.526–3.894)^*^	0.1548
Low CHO intake High CHO intake ^5^	1	1.685 (0.855–3.321) 1.892 (1.146–3.122)	1.748 (0.722–4.236) 3.741 (2.139–6.544) ^***^	0.0083
Low protein intake High protein intake ^6^	1	2.134(1.316–3.459) 1.171(0.554–2.472)	3.370(1.909–5.950) ^***^ 3.229(1.885–5.532)	0.2047
Low fat intake High fat intake ^7^	1	1.743(1.053–2.887) 1.923(0.984–3.760)	3.814(2.589–5.617) ^***^ 2.440(1.076–5.536)	0.1850
Low Na intake High Na intake ^8^	1	1.725 (1.081–2.752) 2.030 (0.914–4.509)	2.751 (1.594–4.749) ^***^ 3.780 (1.542–9.266) ^**^	0.7924
Low BD intake High BD intake ^9^	1	2.244 (1.410–3.896) 0.980 (0.495–1.940)	3.872(2.184–6.863) ^***^ 1.700 (0.730–3.956)	0.0464
Low NBR intake High NBR intake ^9^	1	1.534 (0.946–2.488) 2.524 (1.207–5.281) ^*^	3.325 (1.934–5.717) ^***^ 2.263 (0.903–5.672)	0.1151
Low RD intake High RD intake ^9^	1	1.794 (1.085–2.965) 1.855 (0.945–3.641)	3.477 (1.970–6.139) ^***^ 2.262 (0.997–5.132)	0.4685

Values represent odds ratios and 95% confidence intervals. PRS was divided into three categories (0–3, 4–5, and >6) by tertiles as the low, medium, and high groups of the best model of generalized multifactor dimensionality reduction (GMDR). The reference of cutoff points in each parameter was as following: ^1^ <55 years old, ^2^ <130 mmHg SBP and ≥90 mmHg DBP, ^3^ <126 mg/dL serum glucose concentrations plus hypoglycemic medicine, ^4^ < estimated energy intake, ^5^ <70% carbohydrate (CHO), ^6^ <15% protein, ^7^ <15% fat, ^8^ <1600 mg/1000 kcal Na, and ^9^ <67 percentile of dietary patterns. BD, Balanced diet; NBR, noodle, bread, and red meat diet; RD, rice-rich diet. Multivariate regression models include the corresponding main effects, interaction terms of gene and main effects (energy and nutrient intake), and potential confounders such as BMI, gender, age, smoking, alcohol, education, job, income, energy, physical activity, hypertension, milk, fat percent intake, carbohydrate percent intake, and arthritis and dermatitis medicine intake. Reference was the low-PRS. ^*^ Significantly different from low-PRS in logistic regression analysis at ^*^ *p* < 0.05, ^**^ *p* < 0.01, ^***^ *p* < 0.001.

## Supplementary Materials

The following are available online at https://www.mdpi.com/2072-6643/12/11/3282/s1, Table S1: Generalized multifactor dimensionality reduction (GMDR) results of multi-locus interaction with genes related to glaucoma.
