# Supplementary material for: Polygenetic-Risk Scores for A Glaucoma Risk Interact with Blood Pressure, Glucose Control, and Carbohydrate Intake
Source: Nutrients. 2020 Oct 26;12(11):3282. doi: 10.3390/nu12113282 (PMC7693735; doi:10.3390/nu12113282)
Supplement: Supplementary file 1 [file nutrients-12-03282-s001.pdf]

Table 1. Generalized multifactor dimensionality reduction (GMDR) results of multi-locus interaction with genes related to glaucoma.

GMDR (10 SNPs)	Adjusted for Age, Gender				Adjusted for Age, Gender, Survey Year, Residence Area, BMI			
**Model**	**TRBA**	**TEBA**	**P value**	**CVC**	**TRBA**	**TEBA**	***P* value**	**CVC**
*ABO*_rs2073823	0.547	0.530	8 (0.055)	9/10	0.5458	0.5299	8 (0.055)	9/10
*ABO*_rs2073823<br>*PDE3A*_rs12314390	0.569	0.547	9 (0.011)	7/10	0.5677	0.5415	9 (0.011)	6/10
*CDKN2B*_rs523096<br>*ABO*_rs2073823<br>*CDH13*_rs12449180	0.589	0.574	10 (0.001)	9/10	0.5878	0.5736	10 (0.001)	9/10
*LDB2*_rs3763969<br>*CDKN2B*_rs523096<br>*ABO*_rs2073823<br>*CDH13*_rs12449180	0.607	0.580	10 (0.001)	6/10	0.6055	0.5769	10 (0.001)	5/10
*LDB2*_rs3763969<br>*CDKN2B*_rs523096<br>*ABO*_rs2073823<br>*PDE3A*_rs12314390<br>*CDH13*_rs12449180	0.629	0.594	10 (0.001)	10/10	0.6274	0.5927	10 (0.001)	10/10
*ZFAT*_rs1020236<br>*CDKN2B*_rs523096<br>*ABO*_rs2073823<br>*CDH13*_rs12449180<br>*IGF1R*_rs1319859<br>*SPIRE1*_rs3902981	0.653	0.512	5 (0.623)	3/10	0.6500	0.4989	5 (0.623)	3/10
*LDB2*_rs3763969<br>*ZFAT*_rs1020236<br>*CDKN2B*_rs523096<br>*ABO*_rs2073823<br>*CDH13*_rs12449180<br>*IGF1R*_rs1319859<br>*SPIRE1*_rs3902981	0.684	0.501	4 (0.828)	5/10	0.6803	0.5069	5 (0.623)	3/10
*LDB2*_rs3763969<br>*ZFAT*_rs1020236<br>*CDKN2B*_rs523096<br>*ABO*_rs2073823	0.715	0.507	7 (0.172)	6/10	0.7105	0.5083	6 (0.377)	5/10

*PDE3A*_rs12314390<br>*CDH13*_rs12449180<br>*IGF1R*_rs1319859<br>*SPIRE1*_rs3902981								
*LDB2*_rs3763969<br>*GLI3*_rs1852542<br>*ZFAT*_rs1020236<br>*CDKN2B*_rs523096<br>*ABO*_rs2073823<br>*TRPC4*_rs7335337<br>*CDH13*_rs12449180<br>*IGF1R*_rs1319859<br>*SPIRE1*_rs3902981	0.742	0.516	7 (0.172)	8/10	0.7373	0.5120	7 (0.172)	8/10
*LDB2*_rs3763969<br>*GLI3*_rs1852542<br>*ZFAT*_rs1020236<br>*CDKN2B*_rs523096<br>*ABO*_rs2073823<br>*PDE3A*_rs12314390<br>*TRPC4*_rs7335337<br>*CDH13*_rs12449180<br>*IGF1R*_rs1319859<br>*SPIRE1*_rs3902981	0.765	0.516	7 (0.172)	10/10	0.7603	0.5282	7 (0.172)	10/10

TRBA, trained balanced accuracy; TEBA, test balance accuracy; CVC, cross-validation consistency; sign test, result and P value for the significance of GMDR model by sign test with and without adjusting for covariates designated in the table; BMI, body mass index. *LDB2,* LIM-domain binding protein 2; *CDKN2B,* cyclin-dependent kinase inhibitor 2B; *PDE3A,* phosphodiesterase 3A; *ABO,* alpha 1-3-N-acetylgalactosaminyltransferase and alpha 1-3-galactosyltransferase; *CDH13,* cadherin 13; *GLI3,* GLI family zinc finger 3; *TRPC4,* transient receptor potential cation channel subfamily C member 4; *CDH13,* cadherin 13; *IGF1R,* insulin like growth factor 1 receptor; *SPIRE1,* spire type actin nucleation factor 1.
